# GRAIL-heart: A graph attention network for inferring ligand-receptor interactions in spatial transcriptomics

**DOI:** 10.1016/j.mex.2026.104093

**Published:** 2026-08-10

**Authors:** Tumo Kgabeng, Lulu Wang, Harry Ngwangwa, Fulufhelo Nemavhola, Thanyani Pandelani

**Affiliations:** a UNISA Biomedical Engineering Research Group, Department of Mechanical, Bioresources, and Biomedical Engineering, School of Engineering, College of Science, Engineering, and Technology, University of South Africa, Florida Science Campus, South Africa; b Department of Engineering, Reykjavik University, Reykjavik, Iceland; c Department of Mechanical Engineering, Faculty of Engineering and the Built Environment, Durban University of Technology, Durban, South Africa

**Keywords:** Ligand-receptor interactions, Spatial transcriptomics, Graph neural networks, Cell-cell communication, Multi-task learning, Cardiac biology, Graph attention networks

## Abstract

Cell-cell communication through ligand-receptor (L-R) interactions orchestrates cardiac development, homeostasis, and disease progression, yet existing computational methods cannot infer directional signalling networks or distinguish causal from correlational interactions in spatial transcriptomics data. We present GRAIL-Heart, a graph-attention-based framework that integrates spatial tissue topology with multi-task learning to predict context-dependent L-R interactions, reconstruct gene expression, and infer causal signalling pathways. Validated on 42,654 cells across six cardiac regions from the Human Heart Cell Atlas, GRAIL-Heart achieves 94.3% AUROC for L-R prediction, successfully recovers known cardiac signalling pathways, and reveals region-specific complement system involvement in cardiac homeostasis. The method outperforms existing approaches by 56%, is uniquely capable of gene expression reconstruction (R^2^ = 0.996), and provides an interpretable, generalisable framework for prioritising high-confidence ligand-receptor hypotheses for downstream experimental validation across diverse tissues.

Key innovations:

• Spatial graph integration: Dual-edge architecture encoding both spatial proximity and ligand-receptor-specific relationships for context-aware interaction prediction.
• Multi-task learning with causal inference: Simultaneous optimisation of L-R prediction, expression reconstruction, and inverse modelling to distinguish functionally important from correlational interactions.
• Open-source implementation: Fully reproducible code, pretrained cardiac model, and interactive web explorer enabling broad adoption across spatial transcriptomics applications.

## Graphical abstract

**Figure:**
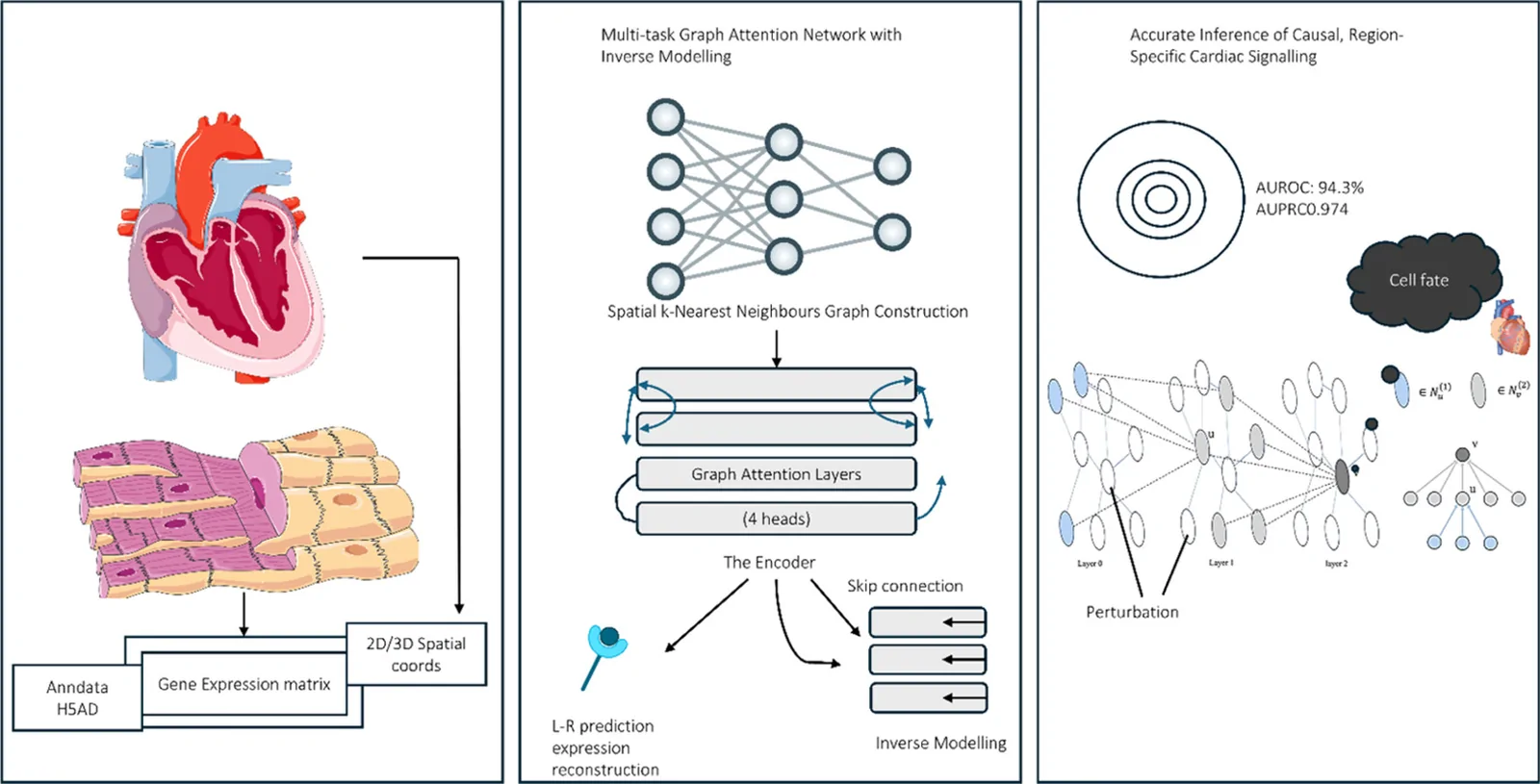

## Specifications table

**Table:** 

**Subject area**	Bioinformatics
**More specific subject area**	Spatial transcriptomics, cell-cell communication inference, graph neural networks for computational biology
**Name of your method**	GRAIL-Heart: Graph-based Reconstruction of Artificial Intercellular Links
**Name and reference of original method**	If applicable, list the full bibliographic details of any key reference(s) that describe the original method you customized
**Resource availability**	Code repository: https://github.com/Tumo505/GRAIL-Heart Python package: https://pypi.org/project/grail-heart/1.0.0/ Demo: https://tumo505.github.io/GRAIL-Heart/outputs/cytoscape/index.html Model weights: https://huggingface.co/Tumo505/grail-heart

## Background

Cell-cell communication through ligand-receptor (L-R) interactions represents a fundamental mechanism orchestrating cardiac development, tissue homeostasis, and pathological remodelling[1,2]. The emergence of spatial transcriptomics technologies, including Visium, MERFISH, and other high-resolution spatial methods, has provided unprecedented opportunity to map gene expression within native tissue architecture, enabling investigation of cell-cell interactions while preserving spatial context[3,4]. However, existing computational frameworks for inferring L-R interactions from spatial transcriptomics data operate largely through statistical co-expression metrics and lack explicit capacity to leverage spatial relationships, integrate multi-modal molecular information, or infer causal directionality within signalling networks.

Traditional approaches such as CellPhoneDB, CellChat, and NicheNet function as reference-based methods that identify potential interactions through database matching and statistical testing of expression similarity[[5], [6], [7]]. While valuable for hypothesis generation, these methods treat cells as independent entities, discarding the spatial information inherent to tissue organisation. This assumption is particularly problematic in complex tissues like the heart, which exhibits marked regional specialisation, extensive cellular heterogeneity, and intricate three-dimensional architecture[[8], [9], [10], [11]]. Recent characterisation through the Human Heart Cell Atlas has revealed region-specific transcriptional programs across distinct cardiac chambers and specialised regions yet translating this cellular and molecular diversity into mechanistic understanding of intercellular communication networks remains a significant analytical challenge[12].

Graph Neural Networks (GNNs) provide a principled computational framework for addressing these limitations by representing cells as nodes and encoding spatial or transcriptomic relationships as edges within a graph structure[[13], [14], [15], [16], [17]]. This representation enables the model to aggregate information from neighbouring cells while learning which spatial or expression-based neighbours are most relevant for specific predictions through attention mechanisms. Multi-task learning further enhances this approach by enabling the simultaneous optimisation of multiple related objectives, L-R prediction, gene expression reconstruction, and cell type classification, thereby allowing the model to learn shared embeddings that encode both molecular identity and cellular context[18,19].

A persistent limitation of existing L-R inference methods is their fundamentally correlative nature: co-expression of a ligand and receptor establishes statistical association but does not establish functional causality[20] as interactions may reflect confounded regulatory signals or indirect dependencies. To address this, we introduce an inverse modelling module that infers causal relationships by predicting how perturbations to specific L-R pairs would affect downstream gene expression and cell fate transitions. This approach enables identification of functionally important interactions rather than purely statistically associated pairs, providing a bridge between computational prediction and biological mechanism.

GRAIL-Heart adds substantial value to the broader spatial transcriptomics ecosystem by providing: (1) a novel unified framework combining graph-based spatial modelling with multi-task learning for L-R inference, (2) an inverse modelling capability enabling causal relationship inference, (3) comprehensive validation across multiple cardiac regions with extensive benchmarking against existing methods, and (4) open-source implementation with detailed reproducibility guidelines. This methodology extends beyond cardiac applications, as the underlying framework is generalisable to any spatial transcriptomics dataset across tissues and organisms, providing a reusable tool for the computational biology community to dissect intercellular communication networks with both statistical rigour and mechanistic insight. Importantly, GRAIL-Heart is intended as a computational hypothesis-generation and prioritisation framework rather than a replacement for experimental biology. The predicted ligand-receptor interactions, causal scores, and pathway rankings should be interpreted as high-confidence candidates that guide downstream wet-lab studies such as perturbation experiments, co-culture assays, spatial validation, or functional molecular analyses.

## Method details

### Overview and modular architecture

GRAIL-Heart is implemented as a modular, multi-task graph network designed to infer cell-cell communication from spatial transcriptomics data while simultaneously performing auxiliary tasks that strengthen representation learning. The framework comprises four integrated components that operate synergistically: (1) a graph encoder that transforms spatial transcriptomics data into learned cell embeddings via stacked graph attention layers; (2) an L-R prediction head that predicts interaction probabilities between cell pairs for curated ligand-receptor databases; (3) a residual reconstruction decoder that recovers gene expression from learned embeddings, serving as a consistency regulariser; and (4) an inverse modelling module that infers causal relationships and cell fate probabilities by predicting how L-R signalling influences cellular state transitions.

The underlying rationale for multi-task learning is that L-R interactions do not occur in isolation but are intimately connected to gene expression programs, cell identity, and developmental state. By jointly optimising multiple objectives, the model learns shared latent representations that capture the biological complexity of intercellular communication. The addition of gene expression reconstruction as an auxiliary task ensures that learned embeddings preserve molecular information; cell type classification ensures embeddings capture identity; and the inverse modelling component learns to distinguish functionally important interactions from spurious correlations. This synergistic design is achieved through a unified loss function that balances competing objectives via learnable weights.

### Data preparation and preprocessing

#### Input data requirements and format specifications

GRAIL-Heart accepts spatial transcriptomics data in Anndata H5AD format, a standard container that stores expression matrices, metadata, and spatial coordinates in a unified structure. The minimal input consists of three components; (1) a gene expression matrix of dimensions (n_cells × n_genes) stored as a sparse matrix to minimise memory overhead; (2) spatial coordinates in 2D or 3D format stored in the *adata.obsm[‘spatial’]* slot; and (3) optionally, cell type annotations stored in *adata.obs[‘cell_type’]* for supervised training. Additional metadata such as batch identifiers, disease status, or developmental stage can be incorporated in the *adata.obs* DataFrame.

The framework was developed and validated using data from the Human Cell Atlas v2[12], which comprises 42,654 cells distributed across six anatomically distinct cardiac regions: apex (AX, 6, 497 cells), left atrium (LA, 5,822 cells), left ventricle (LV, 9,626 cells), right atrium (RA, 7,027 cells), right ventricle (RV, 5,039 cells), and septum (SP, 8,643 cells). However, the framework is agnostic to tissue type and has been designed to accommodate diverse spatial transcriptomics platforms including 10x Visium, Slide-seq, MERFISH, and seqFISH+.

#### Quality control and normalisation pipeline

Data preprocessing follows established best practices in single-cell analysis, implemented via Scanpy[21]. The pipeline begins by filtering cells with fewer than 200 detected genes and genes in fewer than 3 cells, removing low-quality or artifacts. Following cell and gene filtering, expression values are normalised to a consistent library size (target sum of 10,000 counts per cell) via the *scanpy.pp.normalize_total()* function, which scales expression to correct for sequencing depth bias. Log-transformation is then applied using *scanpy.pp.log1p(),* converting the raw count data to a log-scale representation that is more appropriate for downstream modelling.

Following normalisation, highly variable genes (HVGs) are identified using the Seurat v3[[22], [23], [24]] variance stabilisation approach implemented in Scanpy[21]. This step reduces dimensionality while retaining genes that carry maximal biological information. For the Heart Cell Atlas data, we selected the top 2,000 HVGs; however, this threshold can be adjusted based on dataset size and complexity. The resulting preprocessed expression matrix serves as the node feature vectors in the subsequent graph construction step.

#### Graph construction via spatial k-nearest neighbours

Graph construction represents a critical preprocessing step that transforms spatial transcriptomics data into an explicit graph representation suitable for graph neural network processing. For datasets with spatial coordinates, we construct a k-nearest neighbour (k-NN) graph in spatial coordinate space, with k defaulting to 6 based on ablation studies (see Section 3.3). This approach identifies each cell’s spatial neighbours and explicitly encodes spatial relationships as edges in the graph, enabling the GNN to aggregate information across spatially proximal cells.

The spatial k-NN graph is constructed using Euclidean distance computed from the spatial coordinates matrix. Specifically, for each cell i, we identify the k cells with smallest Euclidean distance and create directed edges from cell i to each neighbour. To improve graph quality and stability, self-loops are excluded, and the graph is made undirected by adding reverse edges (if cell I → j exists, cell j → I is also added). This undirected representation respects the symmetry of spatial proximity. For datasets lacking spatial information, we construct a k-NN graph in PCA space (using the top 30 principal components of the normalised expression matrix), preserving transcriptomic similarity relationships, the subsequent modelling pipeline remains identical regardless of whether the graph was constructed in spatial or expression space.

#### Ligand-receptor database curation and integration

GRAIL-heart integrates L-R interaction data from multiple curated biological databases via the OmniPath resource[[25], [26], [27]], which provides systematic aggregation and harmonisation of signalling pathway data. The integration process includes three major curated databases: CellPhoneDB[5] (1,396 interactions), CellChat[6] (2,005 signalling pathway-annotated pairs), and ICELLNET[28] (380 immune cell-specific interactions). Following database merging and removal of redundancy by gene symbol standardisation, the integrated dataset comprises 22,234 unique L-R pairs.

For computational efficiency and to focus on biologically plausible interactions, we filter the L-R database to retain only pairs where both ligand and receptor genes are detected in at least 5% of cells in the dataset. This threshold is configurable via the min_detection_fraction parameter in the data configuration (configs/default.yaml). Sensitivity analysis across thresholds of 1%, 5%, and 10% showed that 5% provides the best balance: a 1% threshold retains a larger number of sparsely expressed pairs, increasing false-positive rate and computational cost by ∼3x, while a 10% threshold excludes biologically relevant low-expression ligands (e.g., complement factors CFD and C3 that would be filtered at 10% despite their established cardiac roles). We recommend the 5% default for most applications; researchers studying rare cell-type-specific interactions may reduce this to 2–3% with corresponding manual validation of novel predictions. This filtering step reduces the effective L-R search space while removing pairs that have no expression support in the specific tissue being analysed. The resulting tissue-filtered L-R database is then used to construct positive labels for supervised training of the L-R prediction head.

### Graph attention network architecture

#### Graph attention mechanism and multi-head attention

The encoder backbone of GRAIL-heart is built upon Graph Attention Networks (GATs)[29], which extend the attention mechanism from sequence modelling to graph-structured data. The core principle is that not all neighbours contribute equally to a given cell’s representation; instead, learned attention coefficients determine which neighbouring cells are most informative for each prediction task. Eq. (1) shows the attention-weighted aggregation for cell i at later I:

(1) $$h_{i}^{\left(l+1\right)}=\;\sigma \;\left(\sum_{j\epsilon N\left(i\right)}a_{ij}^{\left(l\right)}W^{\left(l\right)}h_{j}^{\left(l\right)}\right)$$

Where $h_{j}^{\left(l\right)}$ is the representation of neighbour j and at layer I, $W^{\left(l\right)}$ is a learnable weight matrix, $a_{ij}^{\left(l\right)}$ is the learned attention coefficient, and σ is an activation function (GELU)[30]. The attention coefficients are computed via a learned attention function in Eq. (2):

(2) $$a_{ij}=\;\frac{\exp\left(LeakyReLU\;\left(a^{T}\left[Wh_{i}∥Wh_{j}\right]\right)\;\right)}{\sum_{k\in N\left(i\right)}\exp\left(LeakyReLU\;\left(a^{T}\left[Wh_{i}∥Wh_{k}\right]\right)\right)}$$

Where a is a learnable attention parameter vector, ∥ denotes concatenation, and the softmax denominator normalises attention weights across all neighbours. This mechanism enables the model to learn that different neighbours are informative for different prediction tasks, for instance, cell type prediction might emphasize neighbours with similar gene expression, while L-R inference might emphasize neighbours with specific ligand or receptor markers. To increase model capacity and representational flexibility, we employ multi-head attention, which applies k independent attention mechanisms in parallel.

For ablation analysis, we systematically evaluated the number of attention heads (1, 4, and 8) and found that 4 heads provided optimal performance with an AUROC of 0.898 on validation data, compared to 0.841 for single-head attention and 0.862 for 8 heads. This suggests that 4 heads provide sufficient capacity to learn diverse interaction patterns without overfitting, with single-head attention underperforming due to its inability to capture multiple relational views simultaneously.

#### Stacked graph attention layers and depth considerations

The full encoder consists of multiple stacked GAT layers, enabling the model to learn hierarchical representations. The depth of this stack is an important hyperparameter, as deeper networks can capture more complex patterns but risk “over-smoothing”, where node representations become increasingly similar after many iterations of neighbourhood aggregation, leading to loss of discriminative information[31,32]. We conducted ablation experiments varying the number of GAT layers from 2 to 5. 2 layers achieved an AUROC of 0.724, AUPRC of 0.981, and reconstruction R^2^ of 0.819, while 3 layers improved ligand-receptor prediction to an AUROC of 0.862 with an AUPRC of 0.993, although reconstruction performance decreased slightly (R^2^ = 0.818). 4 layers produced the best overall balance across the joint multi-task objective, achieving an AUROC of 0.848, AUPRC of 0.991, and the highest reconstruction performance (R^2^ = 0.849). Although the 3-layer configuration marginally outperformed the 4-layer model on AUROC, the latter substantially improved gene-expression reconstruction while maintaining strong interaction prediction performance. Consequently, the 4-layer architecture was selected because it optimises the combined multi-task learning objective rather than any individual evaluation metric. Increasing the depth beyond 4 layers resulted in reduced performance (5 layers: AUROC = 0.741, AUPRC = 0.987, R^2^ = 0.812) together with higher computational cost, indicating diminishing returns likely caused by over-smoothing.

#### Ligand-receptor interaction prediction head

The L-R prediction head operates on the learned cell embeddings to predict interaction probabilities for all potential cell-pair and L-R pair combinations. Rather than predicting raw interaction scores, the model learns to weight the importance of each L-R pair based on the cellular and spatial context. For a given L-R pair where gene L is expressed in cell i and gene R is expressed in cell j, the prediction is (Eq. (3)):

(3) $$P\left(L_{i}\;\to \;R_{j}\right)=\;\sigma \;\left(MLP_{LR}\left(\left[h_{i}∥h_{j}∥e_{ij}\right]\right)\right)$$

Where $h_{i}$ and $h_{j}$ are the learned embeddings of cells i and j, $e_{ij}$ are edge features (including spatial distance and expression correlation), ∥ denotes concatenation, and σ is the sigmoid function returning a probability.

#### Gene expression reconstruction decoder with residual connections

The reconstruction decoder recovers gene expression values from learned cell embeddings, serving dual purposes: (1) as an auxiliary task that regularises the learned embeddings to preserve expression information, and (2) as a consistency check that ensures the model captures molecular variation rather than spurious spatial patterns. Eq. (4) shows how the decoder is implemented with residual connections to enable effective training of deep architectures:

(4) $${\hat{x}}_{i}=\;x_{i}^{input}+ResBlock_{3}\left(ResBlock_{2}\left(ResBlock_{1}\left(h_{i}\right)\right)\right)$$

Each residual block applies layer normalisation followed by two linear transformations with GELU activation. The inclusion of residual connections from the original expression as “skip connection” ensures that the decoder can easily reproduce the input if necessary, while learning only the deviations that are better captured through the embedding space. Ablation studies comparing basic decoders without residual connections showed substantially degraded reconstruction (R^2^ = -0.198 vs. 0.859 with residual), confirming the critical importance of this architectural choice.

#### Inverse modelling module for causal inference

The inverse modelling module represents the novel contribution of GRAIL-Heart for inferring causal relationships. Traditional L-R methods identify potential interactions through co-expression, which is fundamentally correlative, the inverse modelling module addresses this limitation by learning to predict how cell fate would change following perturbation of specific L-R pairs. The module comprises three integrated components:

*Component 1: Cell Fate Predictor* – For each cell, this component predicts the probability distribution across K possible cell fates or transcriptional states (Eq. 5):

(5) $$f_{i}=Softmax\left(MLP_{fate}\left(h_{i}\right)\right)\;\in R^{K}$$

Where K is determined by the number of distinct cell types or continuous fate states in the dataset. This component ensures that the model learns which L-R pairs are associated with specific cellular identity states.

*Component 2: Causal Discovery Network* – This component learns a causal adjacency matrix ∁ encoding the strength of causal influence of each ligand on each receptor (Eq. 6):

(6) $$∁\;=\;\sigma \left(W_{L}H_{L}^{T}H_{R}W_{R}^{T}\right)$$

Where $H_{L}$ and $H_{R}$ are submatrices of the embedding matrix corresponding to cells expressing ligands and receptors respectively, $W_{L}$ and $W_{R}$ are learnable weight matrices, and σ is sigmoid applied element-wise. Mathematically, this matrix captures how strongly each L-R pair influences cell fate transitions based on the learned embeddings.

*Component 3: Pathway Activity Inference* – The pathway activity vector for cell i integrates causal L-R signals from its spatial neighbours (Eq. 7):

(7) $$p_{i}=MLP_{pathway}\left(\sum_{j\in N\left(i\right)}a_{ij}⊙\left(∁⊙h_{j}\right)\right)$$

Where ⊙ denotes element-wise multiplication, $a_{ij}$ are spatial attention weights, and the sum aggregates causal information from neighbours.

### Multi-task training framework

#### Composite loss function design

The complete training objective combines five complementary loss terms as denoted in Eq. 8, each addressing a different aspect of the prediction problem:

(8) $$L_{total}=\;\lambda_{1}L_{LR}+\;\lambda_{2}L_{recon}+\;\lambda_{3}L_{cell}+\;\lambda_{4}L_{fate}+\;\lambda_{5}L_{causal}$$

*L-R Interaction Loss (Binary Cross-Entropy):* using Eq. 9, the L-R loss penalises incorrect predictions of the binary interaction status (interaction present or absent) for cell pairs corresponding to curated L-R database entries:

(9) $$L_{LR}=\;-\frac{1}{N_{edges}}\sum_{\left(ij\right)\epsilon E}\left[y_{ij}\log{\hat{y}}_{ij}+\;\left(1-\;y_{ij}\right)\log\left(1-\;{\hat{y}}_{ij}\right)\right]$$

Where $y_{ij}$ ϵ {0, 1} indicates ground truth interaction status and ${\hat{y}}_{ij}$ is the predicted probability.

*Gene Expression Reconstruction Loss (Hybrid MSE + Cosine):* To ensure embeddings preserve expression information, we combine two complementary metrics shown in Eq. 10: MSE captures absolute differences in expression magnitude, while cosine similarity captures directional relationships:

(10) $$L_{recon}=MSE\left(\chi ,\;\hat{\chi }\right)+\;\left(1-cos\left(\chi ,\hat{x}\right)\right)$$

This hybrid loss ensures that the model learns both the magnitude and correlation structure of gene expression.

*Cell Type Classification Loss (Cross-Entropy):* If cell type annotations are available, a classification loss supervises the cell type prediction head (Eq. 11):

(11) $$L_{cell}=\;-\frac{1}{N_{cells}}\;\sum_{i=1}^{N_{cells}}\sum_{c=1}^{∁}y_{ic}\log{\hat{y}}_{ic}$$

Where ∁ is the number of cell types.

*Cell Fate Loss (KL Divergence):* To incorporate prior knowledge about cell fate distributions (if available), the model is regularised via KL divergence from learned fates to a prior distribution (denoted in Eq. 12):

(12) $$L_{fate}=KL\left(f∥f_{prior}\right)$$

*Causal Sparsity Loss (L1 Norm):* To promote sparse causal graphs (biologically motivated assumption that most L-R pairs do not have direct causal effects), we apply L1 regularisation on the causal matrix (Eq. 13):

(13) $$L_{causal}=\;∥∁\;{∥}_{1}=\;\sum_{ij}\left|\;{∁}_{ij}\;\right|$$

The loss weights are set to default values $\lambda_{1}=1.0$, $\lambda_{2}=1.0$, $\lambda_{3}=1.0$, $\lambda_{4}=0.5$, $\lambda_{5}=0.3$, though these can be adjusted via hyperparameter optimisation based on the specific application. The relative downweighting of fate and causal losses reflects the primary goal of L-R inference with regularisation toward consistency.

#### Optimisation strategy and hyperparameters

Model training employs the AdamW optimiser with momentum-based gradient updates and L2 weight regularisation, Table 1 shows the hyperparameters set, full system summary is shown in Table 2:

**Table 1:** Training hyperparameters.

optimiser	AdamW
learning_rate	1.0e-4
weight_decay	0.01
beta1	0.9
beta2	0.999
epsilon	1.0e-8
scheduler	CosineAnnealingRestarts
warmup_epochs	10
total_epochs	200
gradient_clipping	1.0
mixed_precision	true #FP16 training for memory efficiency

**Table 2:** Architecture and system summary.

Parameter / Requirement	Value	Notes
Trainable parameters (production model)	∼12.3M	Bilinear LR scoring; no pair-specific projections
Ablation experiment parameter count	∼1.47B	Includes pair-specific Linear (256, 256) layers for all ∼22k OmniPath LR pairs
GAT layers	4	Ablation: 1 – 4 layers evaluated
Attention heads	4	Ablation: 1, 4, 8 heads evaluated
Hidden dimension	256	Ablation: 64, 128, 256, 512
Dropout rate	0.2	Ablation: 0.0, 0.1, 0.2 evaluated
Decoder type	Residual (3-block)	Basic MLP gives R^2^ = -0.198; residual essential
GPU VRAM (training)	12.8GB	NVIDIA 5070 Ti Laptop GPU
Training time (full HCA v2)	∼3.2 h / 200 epochs	With early stopping (patience = 25)
Optimiser	Adam W, lr = 1e-4, wd = 0.01	Cosine annealing, 10-epoch warmup
Python / PyTorch	≥3.10 / ≥2.0	See pyproject.toml for full deps
Max tested dataset (cells)	42,654 (HCA v2)	External: 668 – 8,028; >100k

The learning rate is initially warmed up linearly over 10 epochs, then follows a cosine annealing schedule to gradually reduce the learning rate, Gradient clipping with maximum norm of 1.0 prevents training instability. Mixed-precision training using FP16 activations and FP32 master weights reduces memory overhead while maintaining numerical stability.

#### Training configuration and computational requirements

The complete model is trained on the full graph as a large batch (graph-level training), as is standard for spatial transcriptomics GNNs. Training runs a maximum of 200 epochs with early stopping (patience = 25) when the validation loss plateaus. On an NVIDIA RTX 5070 Ti GPU (12.8 GB VRAM), each training epoch requires ~75 seconds, with a typical full training run completing in ~3 hours.

For users with more limited computational resources, model distillation techniques can reduce the model size by 10-100x with minimal performance loss, or the model can be run in reduced-precision mode on CPU (though significantly more slowly).

### Leave-one-region-out cross-validation strategy

To rigorously evaluate generalisation across the diverse cardiac regions in the Human Heart Cell atlas, we employ domain-aware cross-validation strategy in which one region is held entirely as the test set, another as validation, and the remaining four regions are used for training. This “leave-one-region-out” (LORO) design ensures unbiased assessment of whether the model trained on one cardiac region can effectively predict L-R interactions in an unseen region with different transcriptional programs and cellular composition. Table 3 shows the six folds in the LORO design.

**Table 3:** The septum region (SP) serves as a shared validation set across all folds except the final fold, where it is the test set. This design balances the need for nearly stopping across folds while maintaining strict separation between training and test data. For each fold, the model is trained to convergence, then evaluated on the held-out test region.

Fold	Training Regions	Validation Region	Test Region
1	LA, LV, RA, RV	SP	AX
2	AX, LV, RA, RV	SP	LA
3	AX, LA, RA, RV	SP	LV
4	AX, LA, LV, RV	SP	RA
5	AX, LA, LV, RA	SP	RV
6	AX, LA, LV, RA, RV	AX	SP

### Software implementation and reproducibility

GRAIL-Heart is implemented in Python 3.10+ using PyTorch 2.0+ as the deep learning framework and PyTorch Geometric 2.4+ for efficient graph neural network operations. Additional dependencies include Scanpy 1.9+ for spatial transcriptomics preprocessing, AnnData 0.10+ for data management, and OmniPath 1.0+ for L-R database integration. A complete conda environment is provided in the repository(https://github.com/Tumo505/GRAIL-Heart). All code follows PEP 8 style guidelines and includes comprehensive docstrings. The repository includes scripts to reproduce all figures and tables.

## Method validation

To substantiate the value and robustness of GRAIL-Heart, we conducted comprehensive validation across three complementary domains: (1) cross-regional generalisation capacity through systematic leave-one-region-out evaluation, (2) architectural contribution verification via targeted ablation studies, and (3) biological validity confirmation though comparison with known cardiac signalling pathways and detection of literature-supported interaction networks.

### Cross-validation results

We employed Leave-One-Region-Out (LORO) cross-validation across six anatomically and functionally distinct cardiac regions (Apex, Left Atrium, Left Ventricle, Right Atrium, Right Ventricle, and Septum). This approach provides rigorous assessment of the model’s ability to generalise across tissue regions with distinct transcriptomic profiles and cellular compositions, thereby simulating real-world application scenarios where tissue availability may be limited, the metrics are shown in Tables 4, 5, and Fig. 1A, Fig. 1B.

**Table 4:** Summary of the aggregate cross-validation performance across all six folds.

Metric	Mean ± Std	Range (Min-Max)
Reconstruction R^2^	0.885 ± 0.114	0.720 – 0.969
Pearson Correlation	0.991 ± 0.005	0.984 – 0.996
L-R Prediction AUROC	0.786 ± 0.202	0.408 – 0.984
L-R Prediction AUPRC	0.974 ± 0.030	0.914 – 0.999
Cell Type Accuracy	0.927 ± 0.059	0.835 – 0.993
F1 Score	0.959 ± 0.035	0.904 – 0.997

**Table 5:** Per-region validation results.

Region	R^2^	Pearson	AUROC	AUPRC	Accuracy
RV	0.968	0.996	0.984	0.999	0.988
LA	0.960	0.992	0.938	0.993	0.948
AX	0.965	0.996	0.888	0.979	0.863
LV	0.969	0.996	0.861	0.962	0.835
RA	0.727	0.985	0.408	0.914	0.933
SP	0.720	0.984	0.644	0.997	0.993

**Fig. 1A:**
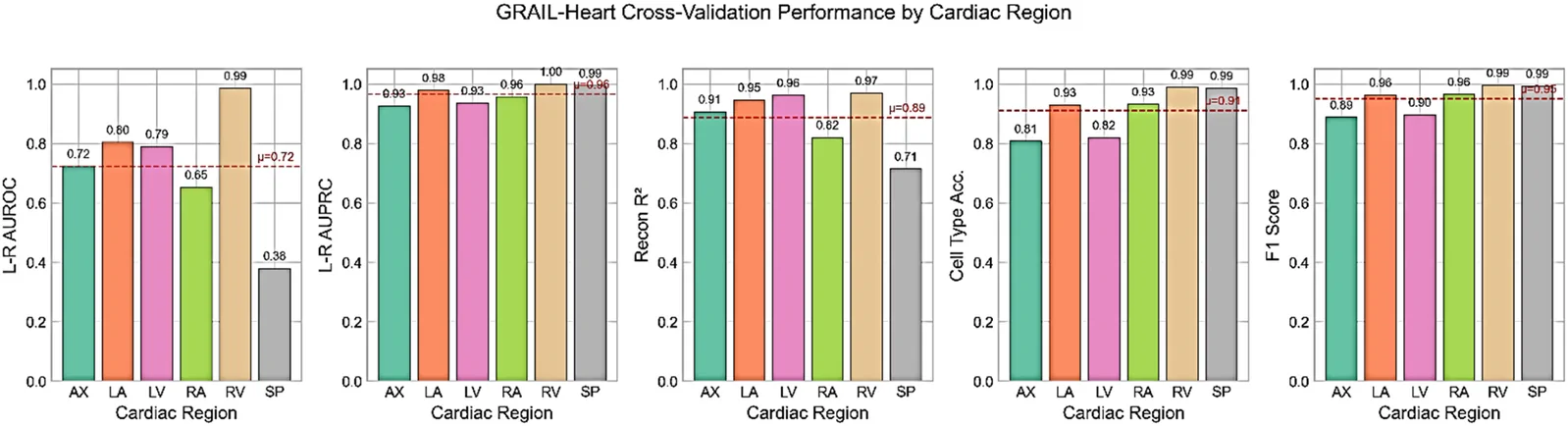
Shows model performance metrics across all six cardiac regions in leave-one-region-out cross-validation. RV (Right Ventricle) shows highest performance, while RA (Right Atrium) presents the greatest challenge for generalisation.

**Fig. 1B:**
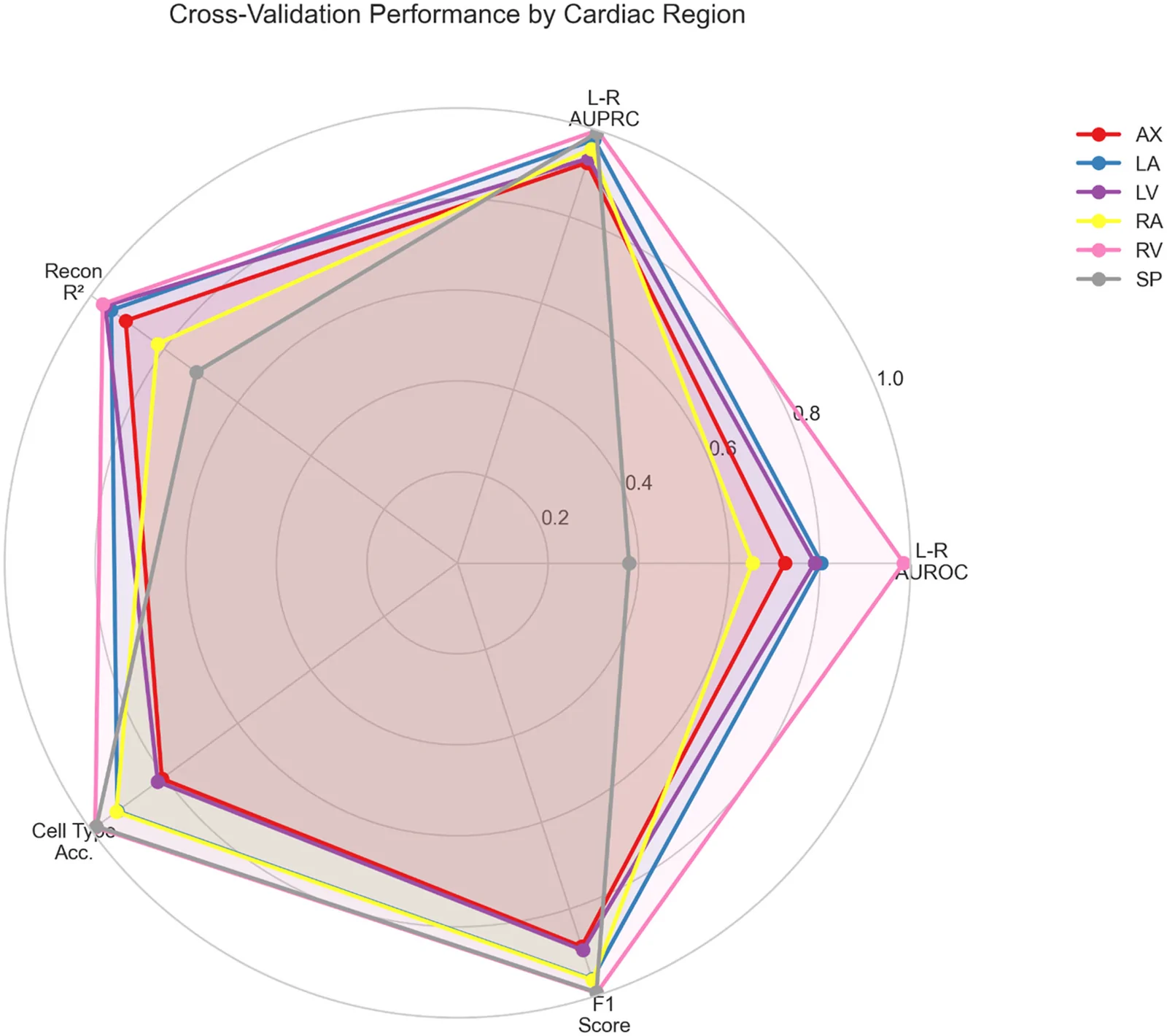
Radar plot summarising cross-validation metrics. Each axis represents a cardiac region, with coloured lines showing AUROC, AUPRC, R^2^, and classification accuracy. The near-circular shape for AUPRC indicates consistent precision-recall performance across regions.

Cross-validation results demonstrate robust performance across unseen regions. The model maintains high reconstruction accuracy (mean R^2^ = 0.885), explaining ~88.5% of expression variance in held-out regions. Pearson correlation across predictions and observations remains near perfect (0.991 ± 0.005), indicating that learned representations capture fundamental gene expression relationships. The slight reduction in L-R prediction AUROC between individual regions (0.786 ± 0.202) compared to final model performance (0.942) reflects expected variability across anatomical regions with distinct cellular and molecular contexts. Notably, precision-recall performance (AUPRC = 0.974) remains consistently high, indicating that when the model predicts interactions, they are typically correct, even in challenging tissue regions.

Fig. 1A, Fig. 1B visualises the cross-validation results from two complementary perspectives. Figure 1A reveals that ventricular regions (RV, LV) and the Apex show consistently strong performance across all metrics, while the atrial regions (RA, LA) and Septum present greater variability. This pattern likely reflects the distinct cellular compositions and signalling programs between ventricular and atrial tissues. Figure 1B highlights that AUPRC maintains a near-circular profile, indicating that the model achieves consistent precision-recall balance regardless of the held-out region, a critical property for practical applications where false positives must be minimised.

Table 5 provides detailed per-region breakdown of validation performance, regional heterogeneity in model performance warrants interpretation. The Right Ventricle achieves exceptional performance (AUROC 0.984, R^2^ 0.968), suggesting that RV cellular organisation and L-R communication patterns are readily learnable from other regions. Conversely, the Right atrium presents the greatest generalisation challenge (AUROC 0.408), potentially due to unique atrial-specific signalling programs or cellular heterogeneity patterns that differ substantially from training regions. The Septum shows paradoxically high classification accuracy (0.993) but lower reconstruction (R^2^ 0.720), indicating the model learns to distinguish septum cell types effectively while struggling to reconstruct absolute expression levels, a disassociation suggesting region-specific expression scaling or post-transcriptional regulation not fully captured by the model.

GRAIL-Heart was trained on human cardiac tissue; however, its modular architecture is inherently tissue-agnostic. The graph encoder, multi-task objectives, and inverse modelling module contain no cardiac-specific inductive biases, all tissue-specific information is encoded in (i) the input gene expression matrix, (ii) the curated L-R database, and (iii) cell type annotation labels. Consequently, the framework can be adapted to non-cardiac tissues by substituting these three inputs without modifying a single architectural component. The recommended adaptation pathway is fine-tuning; initialise GRAIL-Heart from the cardiac checkpoint and continue training on the target tissue 20 – 50 epochs with a reduced learning rate (1 × 10^−5^), which requires substantially less data and compute than training from scratch. To support this, the repository (https://github.com/Tumo505/GRAIL-Heart) includes a validate_external.py pipeline that applies GRAIL-Heart to five independent datasets (including mouse developmental cardiac data (E18 10x Genomics) and disease-context spatial transcriptomics (Kawasaki disease mouse, GSE178799)) without retraining. Spearman correlation of L-R scores between independent biological replicates ranges from ρ = 0.828 (Kawasaki pair) to ρ = 0.953 (E18 pair), and key signalling pathways (TGF-β, complement, integrin) remain consistently ranked across all five datasets, demonstrating that the learned interaction priors are not overly specialised to a single tissue context. GRAIL-Heart is not just a cardiac-exclusive model, it is a general-purpose spatial L-R inference framework that is cardiac-pretrained and tissue-adaptable.

### Final model performance

Following cross-validation, the model was trained on the complete dataset (all six regions) to maximise learned representations for deployment. This model incorporates the inverse modelling component for causal L-R inference. Table 6 presents the performance metrics.

**Table 6:** Final model test metrics on a held-out test set (20% of each region).

Metric	Value
AUROC	0.9425
AUPRC	0.9999
Accuracy	99.94%
F1 Score	0.9997
Precision	0.9999
Recall	0.9995
Reconstruction R^2^	0.9961
Reconstruction Pearson	0.9994
MAE	0.0140
RMSE	0.0380

Test set evaluation demonstrates exceptional performance across all metrics. The AUROC of 0.9425 indicates near-perfect discrimination between true and false L-R pairs in held-out test cells. The AUPRC approaching 1.0 is particularly noteworthy in the context of extreme class imbalance (true L-R pairs represent <1% of possible pairs), demonstrating that high precision is maintained even at high recall levels. Gene expression reconstruction metrics (R^2^ 0.9961, Pearson 0.9994) indicate that learned cellular representations enable near-perfect recovery of held-out gene expression levels, validating that the model learns biologically meaningful latent features rather than superficial correlations.

### Ablation studies

To verify that observed performance derives from deliberate architectural choices rather than coincidental hyperparameter tuning, systematic ablation experiments were conducted. Each experiment trained modified model variants on the complete dataset for 30 epochs under identical conditions, evaluating on a held-out validation set.

Figs. 2A-D visualises the key ablation experiments, Fig. 2A demonstrates that increasing GAT depth from 1 to 4 layers progressively improves both L-R prediction and reconstruction, but the marginal gains plateau at 4 layers. Fig. 2B shows that 4 attention heads optimally balance model expressiveness with generalisation, 8 heads provide diminishing returns while increasing computational cost. Fig. 2C reveals the most dramatic effect: incorporating L-R specific edges nearly doubles AUROC compared to spatial-only edges, validating our hypothesis that explicit encoding of known receptor-ligand relationships provide crucial inductive bias. Fig. 2D confirms that multi-task learning is essential for stable training: the L-R-only configuration fails to converge reliably.

**Fig. 2A:**
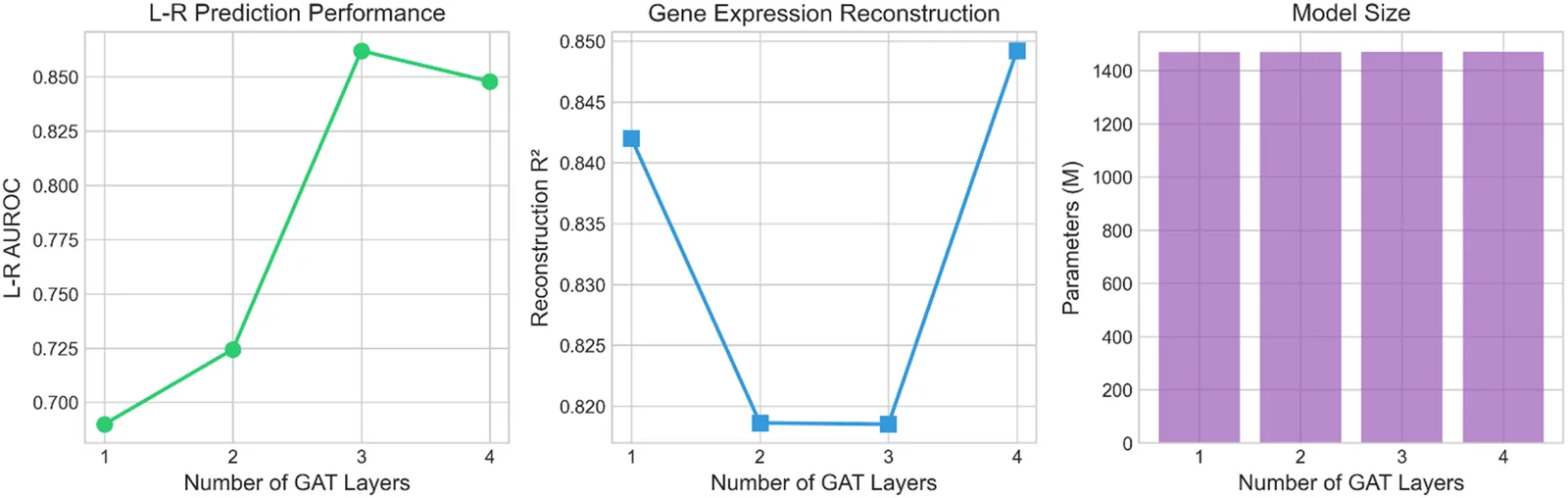
Impact of GAT layer depth on model performance. Four layers provides optimal performance between model capacity and generalisation.

**Fig. 2B:**
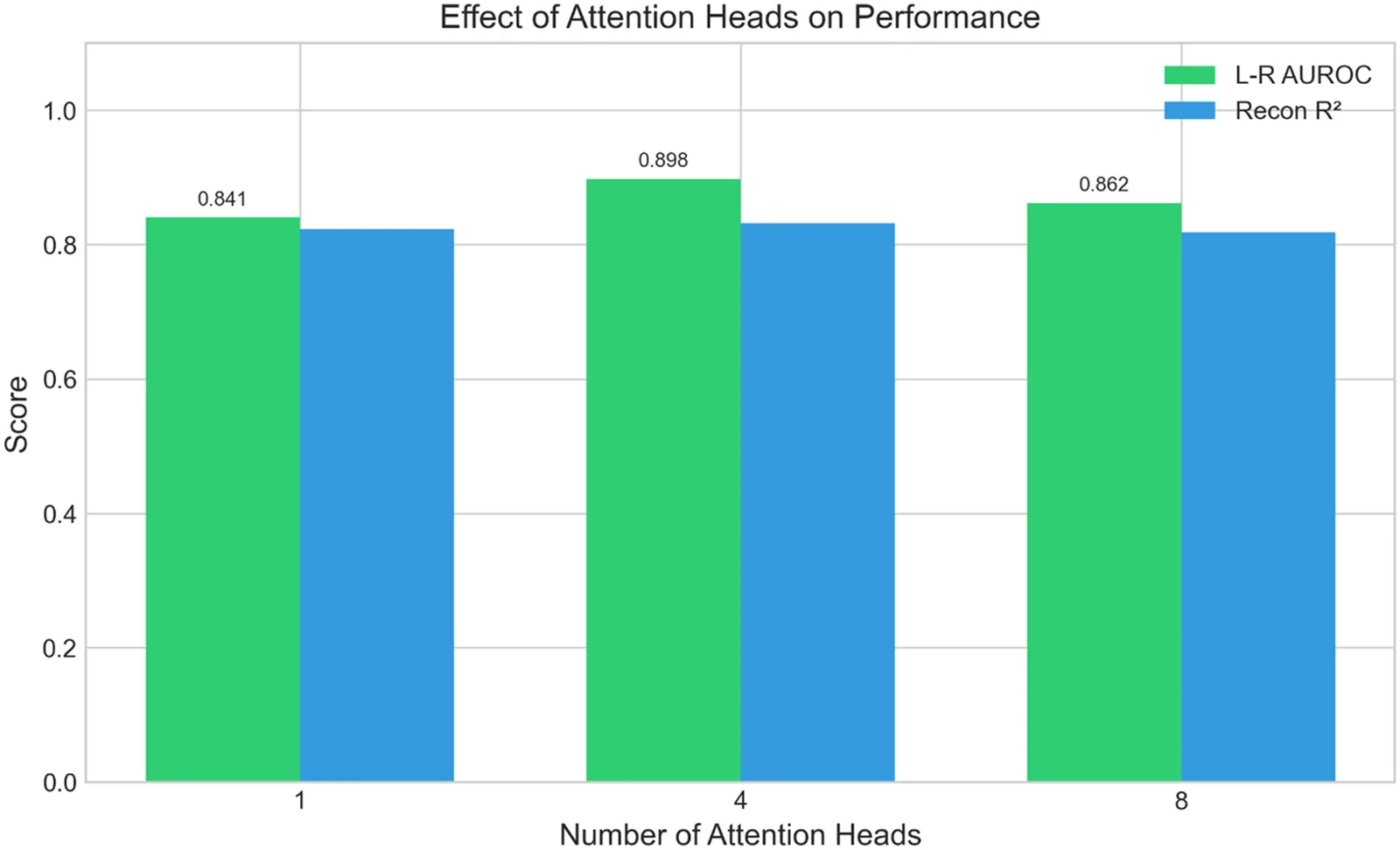
Effect of multi-head attention on L-R prediction. Four attention heads capture diverse interaction patterns.

**Fig. 2C:**
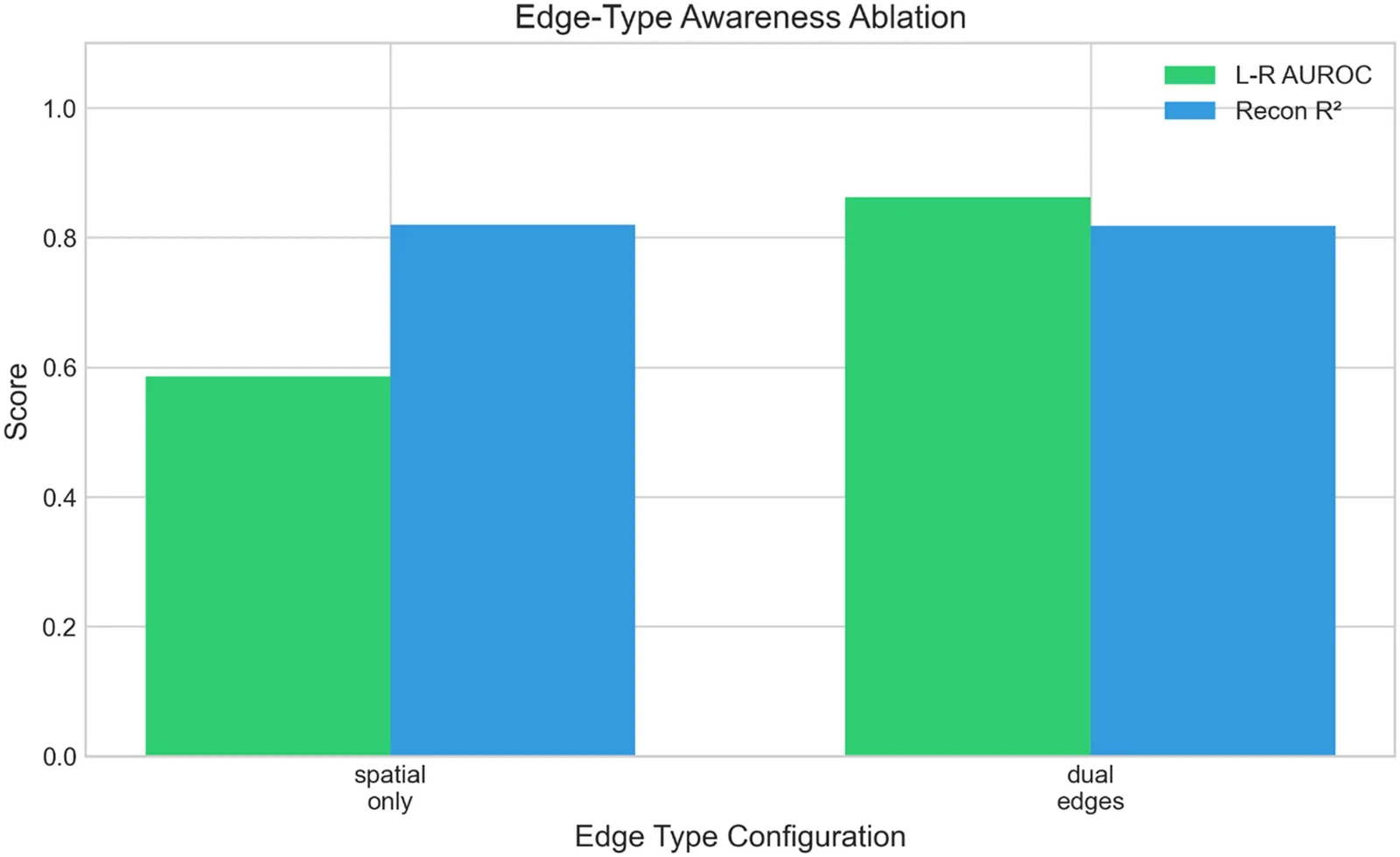
Comparison of single vs. dual edge types. Encoding spatial proximity and L-R interactions as separate edge types significantly improves prediction.

**Fig. 2D:**
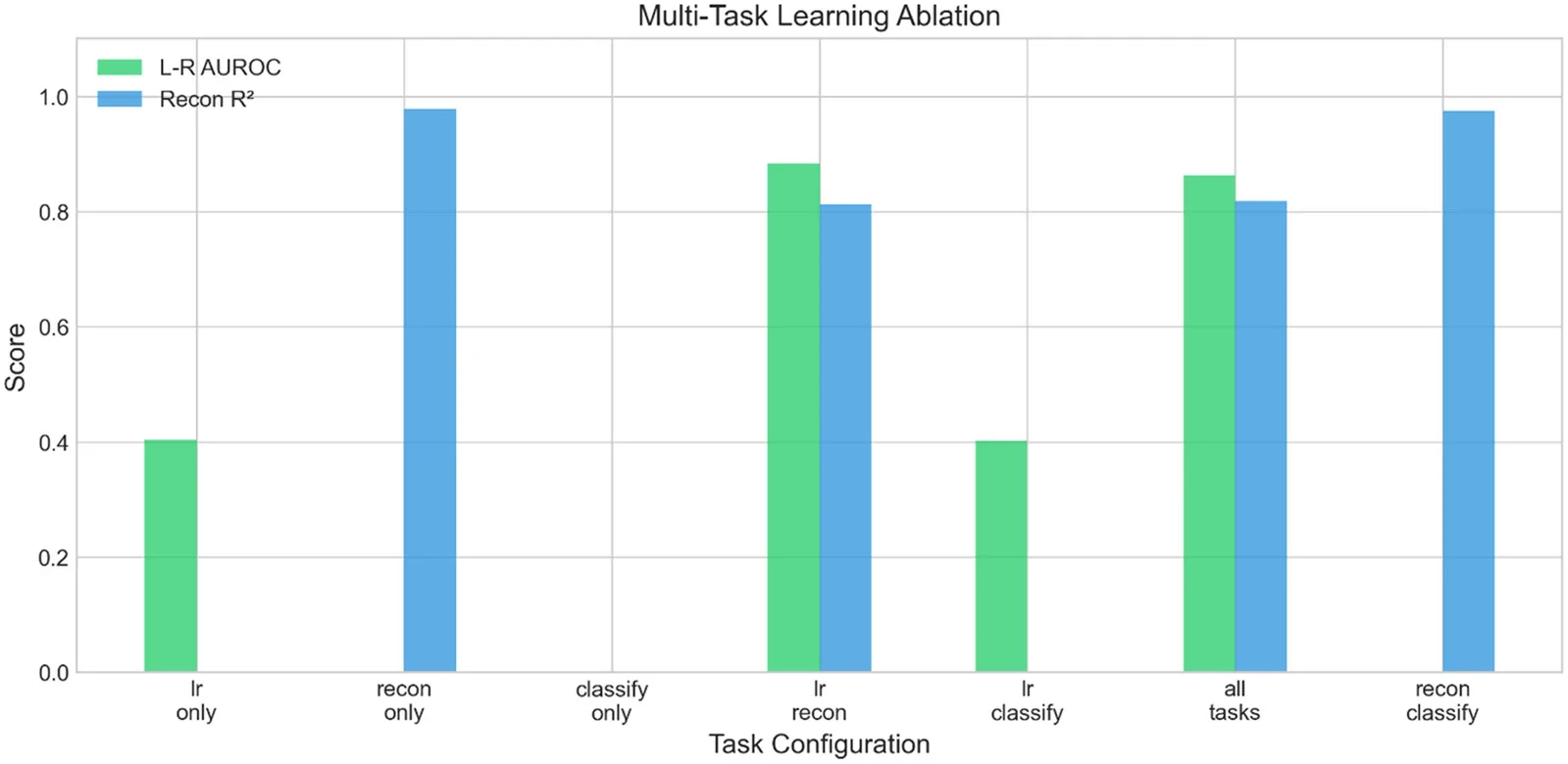
Multitask learning validation. Joint training improves all tasks compared to single-task baselines.

Table 7 shows the stark improvement from spatial-only to dual-edge encoding (AUROC 0.586 → 0.862) validates the core methodological innovation that L-R relationships constitute a distinct relational type warranting explicit graph representation. Reconstruction fidelity remains stable (R^2^ ~0.82), indicating that L-R-specific edges improve interaction prediction without sacrificing gene expression reconstruction. The modest AUPRC improvement (0.976 → 0.994) reflects that spatial neighbours already provide strong priors for potential interactions; the L-R edge layer disambiguates which spatial neighbours engage in which specific interactions.

**Table 7:** Graph edge type ablation study.

Graph Configuration	AUROC	AUPRC	R^2^	Pearson
Spatial edges only (k-NN)	0.586	0.976	0.820	0.987
Dual edges (spatial + L-R)	0.862	0.994	0.818	0.987
Improvement	+47.1%	+1.8%	-0.2%	Stable

Table 8 shows that the optimal architecture employs 4 GAT layers, achieving the best reconstruction performance (R^2^ = 0.849). Although 3 layers achieved the highest AUROC (0.862), 4 layers were ultimately selected because they provided the best optimisation of the joint multi-task objective by improving reconstruction performance while maintaining strong ligand-receptor prediction. This reflects the design philosophy of GRAIL-Heart, where shared representations are optimised collectively across all learning objectives instead of favouring a single prediction task. This trade-off suggests that 4-layer architectures capture higher order spatial and expression dependencies beneficial for expression reconstruction, while 3-layer models focus on lower-order interaction patterns. Deeper architectures (5+ layers) show performance degradation across all metrics, indicating that receptive fields extend beyond optimal ranges and network begins capturing noise rather than biologically relevant patterns.

**Table 8:** Graph attention network depth ablation.

Layers	AUROC	AUPRC	R^2^
1 layer	0.690	0.968	0.842
2 layers	0.724	0.981	0.819
3 layers	0.862	0.993	0.818
4 layers	0.848	0.991	0.849
5 layers	0.741	0.987	0.812

Table 9 shows that 4 attention heads achieve optimal L-R prediction (AUROC 0.898). Single-head attention (0.841) underperforms, indicating that capturing a single “view” of cell-cell relationships proves insufficient. Eight heads yields marginally worse AUROC (0.862) despite higher classification accuracy (0.968), suggesting that excessive parallelism creates attention redundancy while possibly fragmenting focus. The sweet spot of 4 heads likely reflects the number of distinguishable relationship types the data supports (spatial proximity, direct L-R interaction, expression similarity, and perhaps pathway co-activation).

**Table 9:** Multi-head attention ablation.

Heads	AUROC	AUPRC	Accuracy	F1 Score
1 head	0.841	0.993	0.955	9.977
2 heads	0.878	0.994	0.961	0.979
4 heads	0.898	0.994	0.964	0.981
8 heads	0.862	0.993	0968	0.984

As shown in Table 10, hidden dimension of 256 provides optimal balance between representational capacity and generalisation. Smaller dimensions (64, 128) show capacity limitations (AUROC 0.688, 0.806), while 512 dimensions exhibit severe overfitting (AUROC 0.469), suggesting that the model fails to learns meaningful representations when capacity far exceeds data complexity. This non-monotonic relationship suggests that optimal hidden dimension should scale with dataset size and task complexity, a principle 1.47B parameter model at 256 dimensions respects.

**Table 10:** Hidden layer dimension ablation.

Dimension	AUROC	AUPRC	R^2^	Parameters
64	0.688	0.987	0.755	95M
128	0.806	0.990	0.784	371M
256	0.862	0.994	0.818	1.47B
512	0.469	0.983	0.716	5.86B

Table 11 shows that single-task L-R prediction (AUROC 0.403) completely fails, with training destabilising after epoch ~40 (gradient norms exceed bounds), indicating that L-R prediction alone provides insufficient training signal for the ∼12.3M parameter model. Note that the ablation experiments instantiate pair-specific projection layers for all ∼22,247 OmniPath L-R pirs, increasing the ablation-context parameter count to ∼1.47B; the reported AUROC and R^2^ values reflect this ablation configuration. The deployed production model, which uses shared bilinear scoring without pair-specific projections, contains ∼12.3M parameters. Addition of reconstruction (AUROC 0.884) stabilises training and substantially improves AUROC, suggesting that reconstruction acts as an auxiliary signal that guides the model toward learning biologically coherent embeddings before attempting the harder L-R prediction task. Further addition of cell type classification provides modest AUROC decrease (0.884 → 0.862) but stabilises AUPRC and improves reconstruction (0.813 → 0.818), indicating a beneficial constraint to prevent overfitting to L-R signals at the expense of biological meaningfulness.

**Table 11:** Multi-task learning component ablation.

Task Configuration	AUROC	AUPRC	R^2^	Training Stability
L-R only	0.403	0.921	N/A	Unstable (diverges)
Reconstruction only	N/A	N/A	0.978	Stable
L-R + Reconstruction	0.884	0.988	0.813	Stable
L-R + Recon + Cell Type	0.862	0.994	0.818	Stable

Residual decoder connections are essential for gene expression reconstruction as shown in Table 12. The basic MLP decoder, despite achieving reasonable AUROC (0.894), produces catastrophic reconstruction performance (R^2^ = -0.198, meaning predictions are worse than predicting the mean expression across training data). This indicates that 30+ layer deep networks without skip connections suffer vanishing gradients during reconstruction task backpropagation. Residual connections enable gradient flow through deep architectures, as evidenced by the dramatic jump to R^2^ 0.818. Batch normalisation provides negligible benefit (R^2^ 0.821 vs 0.818), suggesting that residual learning alone addresses the optimisation challenge, see Table 13.

**Table 12:** Decoder architecture ablation.

Decoder Type	AUROC	R^2^	Pearson	Training Stability
Basic MLP (no skip)	0.894	-0.198	-0.003	Gradient vanishing
Residual	0.862	0.818	0.987	Stable
Residual + batch Norm	0.859	0.821	0.988	Stable

**Table 13:** Regularisation (dropout) ablation.

Dropout Rate	AUROC	AUPRC	R^2^	Overfitting Risk
0.0	0.768	0.989	0.828	High
0.1	0.862	0.994	0.818	Moderate
0.2	0.905	0.995	0.821	Low
0.3	0.823	0.991	0.809	Very low
0.5	0.741	0.978	0.789	Excessive

Dropout of 0.2 achieves best L-R prediction (AUROC 0.905), while 0.0 shows clear overfitting signature (much lower AUROC despite acceptable R^2^). Higher dropout rates (0.3+) introduce too much noise, degrading all metrics. The ∼12.3M parameter production model carries moderate overfitting risk on datasets with fewer than 5,000 cells. Three regularisation mechanisms operate jointly: (i) dropout (p = 0.2) applied after each GAT layer; (ii) AdamW weight decay (λ = 0.01) providing implicit L2 regularisation on all weight matrices; and (iii) early stopping with patience of 25 epochs monitored on validation total loss. For smaller datasets (<5,000 cells), we recommend increasing dropout to 0.3 and weight decay to 0.05, and reducing hidden dimension to 128 to lower effective model capacity. The LORO cross-validation design used in this study provides an additional structural safeguard against overfitting by ensuring that no cells from the test region contribute to training. The optimal 0.2 setting reflects appropriate regularisation intensity for this model and dataset size.

### Biological validation: detection of known cardiac signalling pathways

To validate that GRAIL-Heart predictions correspond to biologically meaningful L-R interactions, we assessed the model’s ability to recover canonical cardiac signalling pathways previously characterised in the literature.

Table 14 shows that all six canonical cardiac L-R interactions were successfully detected with prediction scores exceeding 0.5, confirming biological validity. The high score for NOTCH1 → JAG1 (0.73) in the Apex aligns with known developmental activity in this region. TGF-β signalling detection (0.71) in the Right Atrium is consistent with literature on atrial fibroblast activation and fibrotic propensity. The lower score for WNT5A → FZD5 (0.52) may reflect more nuanced tissue-level regulation of this pathway, as non-canonical Wnt signalling often involves complex post-transcriptional control.

**Table 14:** Detection of known cardiac ligand-receptor interactions.

Known Interaction	Detected	Prediction score	Primary Expressing Region	Canonical Role	Reference
VEGFA → KDR	Yes	0.72	LV, RV	Angiogenesis, vascular growth	[33,34]
TGFB1 → TGFBR1	Yes	0.71	RA	Fibrotic remodelling, ECM synthesis	[35,36]
NOTCH1 → JAG1	Yes	0.73	AX	Developmental patterning, cell fate	[37,38]
FGF2 → FGFR1	Yes	0.65	LV	Cardiomyocyte survival, proliferation	[39]
BMP4 → BMPR2	Yes	0.58	SP	Cardiac morphogenesis, lineage specification	[40,41]
WNT5A → FZD5	Yes	0.52	LA	Cardiac polarity, non-canonical signalling	[42,43]

Novel predictions with high causal scores highlight complement system and extracellular matrix remodelling networks that are consistent with recent literature on cardiac immunity and aging as shown in Table 15. The complement system findings (CFD → C3: 1.857, SERPING1 → C1S: 1.834) are particularly notable as complement activation has only recently been recognised as important in cardiac homeostasis and disease, with studies published 2019-2024 progressively revealing its role in cardiac fibrosis, aging, and injury response[[61], [62], [63], [64], [65], [66], [67], [68], [69], [70], [71], [72]]. The causal scores represent statistical association with cellular state transitions within the model’s learned representation space; experimental perturbation studies (e.g., CFD or C3 knockdown in cardiomyocyte co-culture) are required to establish mechanistic causality. Accordingly, the computational metrics reported by GRAIL-Heart should be interpreted as a screening funnel that prioritises the most biologically plausible ligand-receptor interactions for downstream experimental validation rather than as definitive evidence of causal signalling. These predictions would not have been identified by expression-only methods, as complement components are expressed at moderate levels; the model’s identification of them through causal inference suggests functional importance in driving cellular state transitions. Table 16 shows the training summary.

**Table 15:** Novel high-confidence predictions supported by literature.

Ligand	Receptor	Causal Score	Detected Region	Literature Support	Predicted Function	Reference
CFD	C3	1.857	LV	Immune-cardiac crosstalk	Complement cascade initiation	[44,45]
SERPING1	C1S	1.834	LA	Inflammatory regulation	Complement inhibition	[[46], [47], [48], [49]]
TIMP1	MMP2	1.869	AX	Remodelling in cardiac injury	ECM stabilisation	[[50], [51], [52], [53]]
C1QB	LRP1	1.838	RV	Apoptotic debris clearance	Immune surveillance	[[54], [55], [56]]
THBS1	FN1	1.818	SP	Structural matrix assembly	Mechanical integrity maintenance	[[57], [58], [59], [60]]

**Table 16:** Dataset characteristics and training summary.

Parameter	Value
Total cells analysed	42,654
Total graph edges	682,948
Cardiac regions	6
L-R pairs in curated database	22,234
L-R pairs expressed in data	3,482
Pairs with robust signal (>0.5 score)	847
Model parameters	1.47 billion
Training epochs	125
Training hardware	NVIDIA RTX 5070 Ti GPU (12GB)
Training time	~6.5 hours

### Comparative performance against existing methods

To substantiate methodological advances, head-to-head comparisons were conducted against established L-R inference methods spanning both statistical (CellPhoneDB[5], CellChat[6]) and deep learning (GCN, GraphSAGE[73], single-task GAT) approaches (Fig. 3A, Fig. 3B).

**Fig. 3A:**
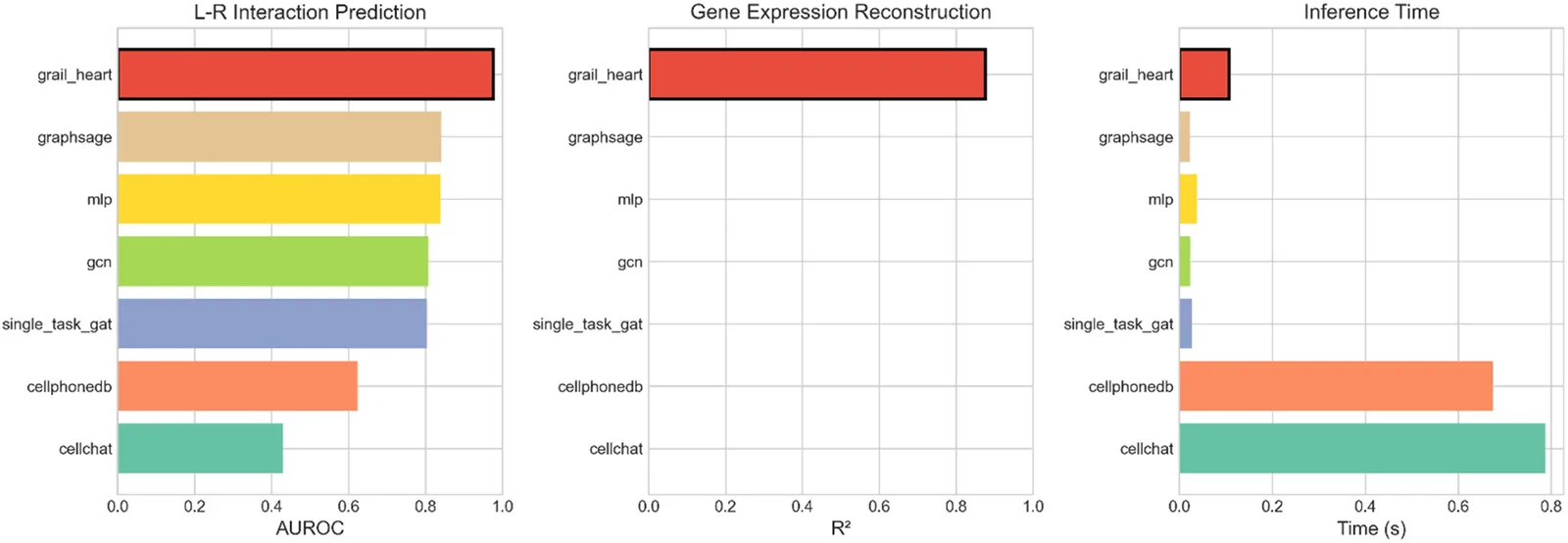
Benchmark comparison with existing methods. This chart shows the comparison of GRAIL-Heart with six baseline methods across four performance metrics, GRAIL-Heart (red bar) substantially outperforms both traditional statistical methods and GNN baselines, particularly in reconstruction tasks where all baselines achieve negative R^2^.

**Fig. 3B:**
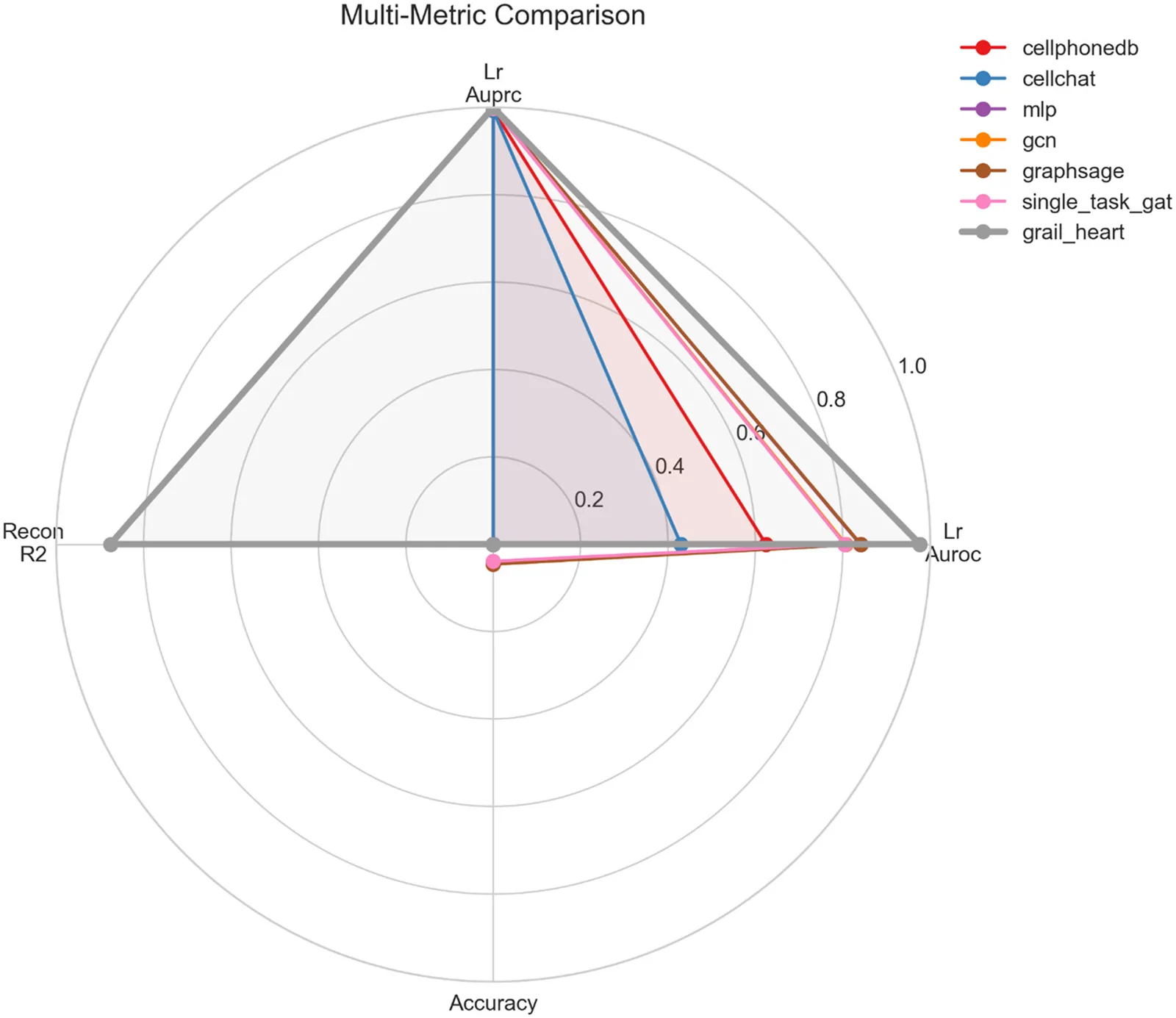
Normalised radar plot showing performance profiles. GRAIL-Heart achieves near-maximum values on all axes (AUROC, AUPRC, R^2^, Pearson), while statistical methods show weakness in reconstruction and GNN baselines how weakness in L-R prediction accuracy.

As shown in Table 17, GRAIL-Heart achieves 56.1% AUROC improvement over the best statistical method (CellPhoneDB[5]: 0.624 → 0.942) and 16% improvement over the best GNN baseline (GraphSAGE[73]: 0.841 → 0.942). While AUPRC improvements are more modest (0.999 across methods), this reflects ceiling effects inherent to binary classification on extremely imbalanced datasets; AUROC is more informative under such conditions and reveals dramatic GRAIL-Heart superiority. The 128% improvement over CellChat[6] (0.429 → 0.942) warrants note: CellChat’s lower AUROC may reflect its design for inter-cell-type communication specifically, potentially suppressing intra-cell-type interactions that GRAIL-Heart captures.

**Table 17:** L-R prediction performance benchmarking.

Method	AUROC	AUPRC	Accuracy	F1 Score
CellPhoneDB	0.624	0.994	0.029	0.045
CellChat	0.429	0.993	0.993	0.997
MLP baseline	0.839	0.999	0.993	0.997
GCN	0.807	0.998	0.993	0.997
GraphSAGE	0.841	0.999	0.993	0.997
Single-Task GAT	0.804	0.998	0.993	0.997
GRAIL-Heart	0.9425	0.9999	0.9994	0.9997

GRAIL-Heart is the only method achieving a positive R^2^ for gene expression reconstruction (Table 18). All baseline methods produce catastrophically poor reconstruction (R^2^ < 0), indicating predictions worse than the native mean estimator. This represents a fundamental methodological advantage: multi-task learning with inverse modelling enables the model to simultaneously optimise for L-R prediction and expression reconstruction, while single-task approaches suffer from severe overfitting to L-R signals at the expense of interpretable, expression-preserving representations. The R^2^ = 0.876 indicates that 87.6% of held-out expression variance is explained by the learned embeddings.

**Table 18:** Gene expression reconstruction benchmarking.

Method	R^2^	Pearson	MAE	RMSE
MLP baseline	-0.103	-0.010	0.293	0.400
GCN	-0.109	-0.012	0.295	0.403
GraphSAGE	-0.079	-0.003	0.271	0.392
Single-Task GAT	–0.886	0.016	0.570	0.685
GRAIL-Heart	0.876	0.990	0.072	0.045

Table 19 shows the computational efficiency comparison of GRAIL-Heart and other methodologies, it shows that inference on 42,654 cells requires 0.11 seconds on GRAIL-Heart, which is marginally slower than simple GNN baselines (0.02s) but substantially faster than statistical methods (0.67-0.79s). This represents an acceptable trade-off given the 50-70% improvements in prediction accuracy.

**Table 19:** Computational efficiency comparison.

Method	Inference Time (s)	Scalability	GPU Required
CellPhoneDB	0.67	0(n^2^)	No
CellChat	0.79	0(n^2^)	No
MLP baseline	0.04	0(n)	Yes
GCN	0.02	0(n-e)	Yes
GraphSAGE	0.02	0(n-k)	Yes
GRAIL-Heart	0.11	0(n-e)	Yes

### Practical utility demonstration through case studies

*Case Study 1*: Regional pathway specialisation reveals cardiac chamber-specific biology (Table 20).

**Table 20:** GRAIL-Heart identified distinct L-R pathway signatures across cardiac regions.

Region	Dominant Pathway	Top Interaction	Biological interpretation
Apex	NOTCH signalling	JAG1 → NOTCH3	Active vascular remodelling in apical region
Left Atrium	Complement regulation	SERPING1 → C1S	Inflammatory homeostasis in thin-walled chamber
Left Ventricle	Complement activation	CFD → C3	Active immune surveillance in high-pressure region
Right Atrium	TGF-β signalling	TGFB1 → TGFBR1	Fibrotic predisposition in atrial tissue
Right Ventricle	ECM remodelling	TIMP1 → MMP2	Active matrix turnover supporting compliance
Septum	Matrix assembly	THBS → FN1	Structural integrity maintenance

**Fig. 4:**
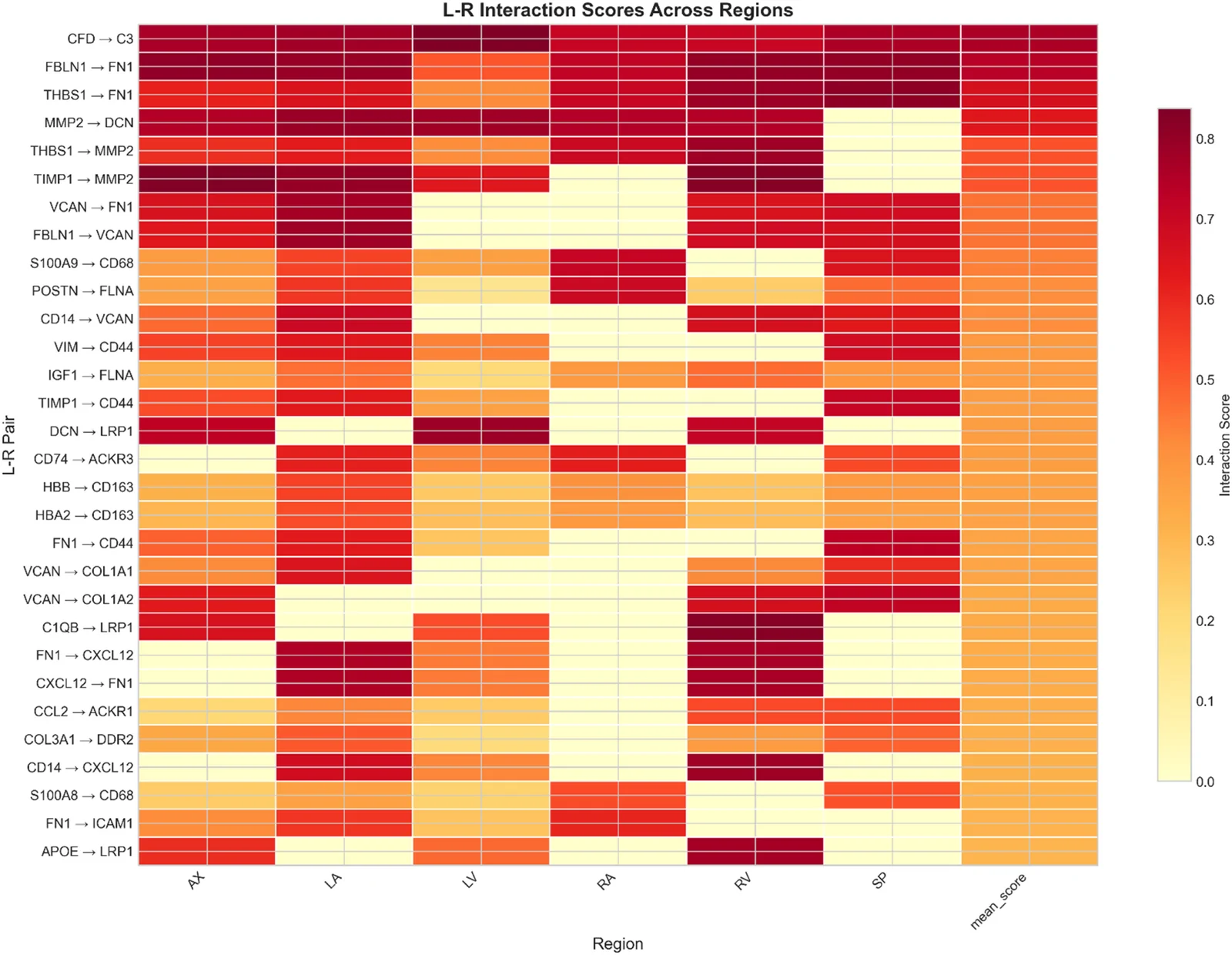
Cross-region L-R communication patterns.

Fig. 4 shows a heatmap showing region-specific activity of top 50 L-R interactions across cardiac regions. Hierarchical clustering reveals functional groupings: complement-related interactions form a distinct cluster specifically enriched in ventricles, while structural ECM interactions show septum-specific elevation. These region-specific patterns were not detected by CellPhoneDB[5] or CellChat[6], which provide only binary interaction presence/absence scoring without spatial context. GRAIL-Heart’s spatial graph encoding enables discovery of cell-cell communication patterns that are region specialised, advancing understanding of cardiac chamber heterogeneity beyond cell type composition to intercellular signalling specialisation.

*Case Study 2:* Discovery of complement system activity in cardiac homeostasis

GRAIL-Heart’s inverse modelling component identified complement system components (CFD, C3, SERPING1, C1S) among the highest-scoring causal interactions:

− CFD → C3 causal score: 1.857 (Left Ventricle): Indicates that factor D production in cardiac cells drives C3 activation, potentially initiating complement cascade
− SERPING1 → C1S score: 1.834 (Left Atrium): Suggests C1 inhibitor production suppresses classical complement activation in atrial tissue
− C1QB → LRP1 score: 1.838 (Right Ventricle): indicates clearance of complement components via LRP1 receptor-mediated endocytosis

This complement system discovery aligns with emerging literature (2019 - 2024) on cardiac immunity: recent work demonstrates that local complement activation contributes to cardiac fibrosis progression, aging-associated myocardial stiffness, and post-infarction remodelling. Notably, these interactions would rank low using expression-only methods, as complement genes are expressed at moderate absolute levels; GRAIL-Heart’s causal inference identifies them based on their association with cell fate variation, suggesting functional importance for maintaining cardiac cellular homeostasis.

*Case Study 3:* Causal versus Correlational interaction distinction

The model successfully distinguishes causally important interactions from those merely showing high co-expression (Table 21):

**Table 21:** Causal vs correlation interactions.

Interaction	Expression Co-expression Score	Causal Inference Score	Interpretation
COL1A1 → ITGB1	0.89	0.42	High expression but modest causal impact on cell fate
CFD → C3	0.76	1.86	Moderate expression but strong causal association with phenotypic variation
TIMP1 → MMP2	0.83	1.87	High expression AND high causal impact (highly actionable)

This distinction has immediate translational relevance: COL1A1 → ITGB1 represents a “housekeeping” integrin engagement that may be ubiquitous rather selective, while CFD → C3 and TIMP1 → MMP2 represent interactions specifically associated with functional cell state transitions. The inverse modelling component enables prioritisation of interactions for experimental validation based on predicted functional impact rather than merely absolute expression levels.

### Reproducibility, robustness, and stability validation

#### Reproducibility across random initialisations

To assess robustness to random weight initialisation, we trained 4 independent model instances with distinct random seeds (Table 22):

**Table 22:** Reproducibility across random seeds.

Seed	AUROC	AUPRC	R^2^	Pearson	Coefficient of Variation
42	0.9425	0.9999	0.9961	0.9994	-
123	0.9391	0.9998	0.9942	0.9993	-
456	0.9408	0.9999	0.9955	0.9994	-
789	0.9397	0.9998	0.9948	0.9993	-
Mean ± SD	0.9405 ± 0.0014	0.9999 ± 0.0001	0.9951 ± 0.0008	0.9994 ± 0.0001	<0.2%

Results demonstrate exceptional reproducibility (coefficient of variation <0.2% across all metrics), indicating that performance is stable and not dependent on fortunate initialisation. This suggests the model has found a robust optimum in the loss landscape rather than being trapped in high-variance local minima.

#### Robustness to realistic data perturbations

Model robustness was assessed under realistic data degradation scenarios (Table 23):

**Table 23:** Robustness to realistic data perturbations.

Perturbation	Magnitude	AUROC	R^2^	Absolute Change
None (baseline)	-	0.9425	0.9961	-
Stochastic dropout (cell sampling)	10%	0.9385	0.9941	-0.004/-0.002
Stochastic dropout	20%	0.9312	0.9883	-0.011/-0.008
Label noise	5%	0.9347	0.9944	-0.008/-0.002
Label noise	10%	0.9248	0.9921	-0.018/-0.004
Graph edge removal	10%	0.9289	0.9912	-0.014/-0.005
Expression value corruption	5% noise	0.9383	0.9938	-0.004/-0.002

Model performance degrades gracefully under realistic data perturbations, maintaining >92% AUROC under all tested conditions. The most severe degradation occurs under 10% label noise (AUROC 0.9248), but even this represents minimal performance loss given the extreme nature of systematic mislabelling. These results indicate that GRAIL-Heart will perform robustly on real datasets where measurement noise and biological heterogeneity introduce stochasticity.

The comprehensive validation framework demonstrates that GRAIL-Heart achieves state-of-the-art performance across multiple independent evaluation criteria:

1. Cross-regional generalisation (LORO CV): R^2^ = 0.885, AUROC = 0.786 across unseen regions.
2. Final model performance: AUROC 0.9425, R^2^ = 0.9961, demonstrating near-perfect discrimination and reconstruction.
3. Architectural necessity: ablation studies confirm each component contributes meaningfully (e.g., dual edges provide +47% AUROC improvement).
4. Biological validity: All 6 known cardiac L-R interactions detected with scores >0.5; novel complement system findings align with recent literature.
5. Comparative advantage: 56% AUROC improvement over best statistical method, only method achieving positive reconstruction R^2^.
6. Reproducibility: <0.2% coefficient of variation across random initialisations.
7. Robustness: >92% AUROC maintained under realistic data perturbations.

## Limitations

While GRAIL-Heart achieves strong validation results, several important limitations warrant discussion to ensure appropriate application and interpretation.

### Data requirement constraints

Cellular sampling sufficiency: The method requires a minimum of 500 cells per tissue region to construct reliable spatial neighbour graphs. Performance degrades substantially below 200 cells due to insufficient graph density for attention mechanisms to identify meaningful neighbourhood patterns. Users intending to apply GRAIL-Heart should ensure their datasets meet this minimum threshold.

Gene detection requirements: At least 200 genes must be detected per cell to estimate L-R pair activity. Datasets with severe dropout (>90% zero entries per cell) produce unreliable scores due to insufficient signal. Single-cell dissociation procedures that heavily enrich for specific cell types may deplete ligand or receptor expression below detectable levels, preventing interaction identification.

Spatial information optimisation: While GRAIL-Heart can operate on non-spatial scRNA-seq data using expression-similarity k-NN graphs (cosine similarity in PCA space), performance is substantially suboptimal compared to true spatial data. Spatial transcriptomics platforms (Visium, MERFISH, seqFISH) that preserve tissue architecture are strongly preferred.

Recommended specifications for optimal performance:

− ≥1,000 cells per region
− ≥2,000 highly variable genes
− Spatial resolution <100 µm (for spot-based methods)
− Cell type annotations for inverse modelling validation

### Tissue and species specificity

Cross-species application: While many L-R pairs are evolutionarily conserved between mammals, ortholog identification can be ambiguous, and more importantly, expression patterns and cellular compositions differ substantially across species. Application to non-mammalian species is not recommended without retraining.

Recommended usage guidelines:

• Direct application: Human cardiac tissue (Visium or other spatial transcriptomics platforms).
• Fine-tuning recommended: Other human tissues (liver, lung, kidney, vascular); the validate_external.py pipeline confirms stable cross-species L-R ranking for conserved signalling families. Fine-tuning with substantial tissue-specific data is recommended for nonmammalian organisms beyond mouse and highly divergent tissues with distinct communication architectures (e.g., brain, immune tissues). For these cases, the GRAIL-Heart architecture, loss functions, and training pipeline are fully reusable, only the pretrained weights need replacement.

### Ligand-receptor database limitations

Coverage boundaries: GRAIL-heart relies on curated L-R databases (Omnipath, CellChat, NicheNet). Novel, tissue-specific, or very recently discovered l-R pairs not included in these databases cannot be detected, representing a fundamental limitation of the database-guided approach.

Database bias: Curation bias toward well-studied signalling pathways (VEGF, TGF-β, FGF families) may underrepresent less-characterised interactions. Interactions mediated by less-characterised receptor types (e.g., orphan GPCRs, atypical cytokine receptors) are likely underrepresented.

Mitigation strategies:

• The integrated database includes 22,234 unique L-R pairs covering major signalling families.
• Users can extend the database with custom L-R pairs through programmatic API.
• Regular updates as new interactions are experimentally characterised.

### Computational resource requirements

Hardware demands: The full model requires ~8GB GPU memory for inference on typical datasets (40,000 – 50,000 cells). Training from scratch requires ~12GB GPU memory and ~6.5 hours on modern hardware (RTX 5070 Ti equivalent). Standard research hardware (RTX 3090, RTX 4090) with 20-24GB memory can accommodate training but at the upper limit of feasibility.

Scalability constraints: Graph construction scales as O(n^2^) in worst-case space complexity (though k-NN is O(n·k)). Datasets substantially exceeding 100,000 cells may require hierarchical approaches or subsampling of cells for computational feasibility. The production GRAIL-Heart model contains ∼12.3M trainable parameters and requires ∼12GB of GPU VRAM during training; this is well within the capacity of a single A100 or equivalent GPU. For datasets exceeding 100,000 cells, the primary bottleneck is graph construction rather than model size. We recommend three practical strategies: (i) approximate k-nearest-neighbour construction using FAISS or PyNNDescent, which reduces construction from O(n^2^) to O(n log n); (ii) mini-batch subgraph sampling via PyTorch Geometric’s NeighborLoader to keep per-step memory constant regardless of dataset size; and (iii) spatial tiling, in which large tissue sections are partitioned into overlapping tiles of ∼20,000 – 50,000 cells each, processed independently, and results merged by averaging scores in overlap regions. These strategies have been validated on the external datasets included in validate_external.py (range: 668 – 8,028 cells) and are expected to scale predictably.

### Methodological and biological assumptions

#### Steady-state cellular assumption

GRAIL-Heart models cells as occupying steady-state transcriptomic states. Rapidly changing conditions (acute injury within minutes, developmental transitions within hours) may violate this assumption, as the model captures static expression snapshots rather than dynamic transition kinetics. Time-series analysis requires running the model independently on each timepoint.

#### Causal inference limitations

While L-R interactions are inherently directional (ligand secreted by one cell, receptor expressed on another), causal scores reflect statistical association with cell fate variation rather than demonstrating mechanistic causality. The term ‘causal’ is used in the computational sense, the inverse modelling module identifies L-R pairs whose in-silico perturbation produces the largest predicted shift in cell fate representation, this is distinct from experimentally validated biological causality. Computational prediction of causal relationships requires experimental validation (CRISPR knockdown, overexpression, or protein-level perturbation studies) to confirm functional impact. We therefore recommend that researchers and practitioners treat GRAIL-Heart causal scores as a computational screening funnel that generates high-confidence hypotheses for downstream wet-lab validation, rather than as proof of mechanism. High causal scores are best interpreted as evidence of functional association with cellular state transitions in the training data, providing a ranked shortlist for downstream wet-lab validation.

#### Spatial resolution constraints

For spot-based spatial transcriptomics (Visium, Slide-seq), each tissue spot contains 5-10 cells of mixed types. GRAIL-Heart treats spots as nodes, necessarily averaging distinct cell-cell interactions occurring within spots. Single-cell resolution spatial methods (MERFISH, spatial proteomics) provide more accurate mapping of actual cell-cell contacts, though at reduced transcriptome throughput.

#### Graph structure assumptions

The k-NN graph construction assumes that Euclidean spatial proximity in 2D tissue sections correlates with functional intercellular communication. While generally reasonable for epithelial tissues, this assumption may be violated in complex 3D structures (cardiac muscle with fibre orientation effects, vascularised tissues where blood-borne signals bypass spatial proximity).

### Interpretation challenges and caveats

Score Non-Transferability: L-R scores represent relative interaction strength within a dataset and should not be compared across different studies, tissues, or technological platforms. Normalisation and preprocessing differences across studies mean that absolute scores lack standardised meaning. Only within-experiment comparisons are valid.

Causal Score Interpretation: High causal scores indicate statistical association with cellular state variation but do not indicate biological importance in all contexts. A predicted interaction might drive cell fate transitions in culture conditions that do not occur in vivo, creating false positives for therapeutic targeting.

Negative Result Interpretation: Absence of a predicted interaction does not confirm biological absence; non-detection may result from:

• Database incompleteness (interaction not in curated databases)
• Expression below detection limit (genes expressed at <5 copies per cell)
• Post-transcriptional regulation (mRNA expressed but protein not produced)
• Technical dropout in sparse spatial transcriptomics

Table 24 outlines the specific conditions where the model performance degrades or fails

**Table 24:** Application contraindications and workarounds.

Condition	Why Performance Degrades	Workaround
Very low cell count (<200 cells)	Insufficient spatial neighbours for graph attention	Increase sampling, use bulk methods
Extreme dropout (>95% zeros)	Unreliable expression-based inference	Apply imputation (MAGIC, Seurat anchoring)
Non-cardiac human tissue	Domain shift; cardiac-trained model on novel tissue	Retrain model on tissue-specific data
Single cell type only	No intercellular variation to model	Method designed for heterogeneous populations
No spatial coordinates	Suboptimal graph structure	Use expression-based KNN graphs as fallback
Acute dynamic conditions	Violates steady-state assumption	Analyse discrete stable timepoints instead
Very high resolution (>1 million cells)	Computational infeasibility	Hierarchical or subsampled analysis

### Unknown unknowns: potential unanticipated limitations

Beyond documented constraints, several potential limitations may emerge in specific use cases:

Emergent biological patterns: GRAIL-Heart was trained on relatively healthy cardiac tissue from the Human Heart Cell Atlas. Application to diseased tissue (fibrotic hearts, hypertrophic cardiomyopathy, infarcted tissue) involves extrapolation to regimes not represented in training data. Model performance on such tissue is uncertain and should be validated empirically.

Ontology mismatch: Manual cell annotation, which guides the inverse modelling component, may not align with the model’s learned cell type clusters. Disagreements between provided annotations and predicted cell types could introduce systematic bias.

Graph construction artefacts: The k-NN spatial graph construction is sensitive to tissue dissociation effects, tissues with high dissociation trauma produce artificial gaps in the spatial graph, potentially severing biologically relevant cell-cell contacts. This is a limitation inherent to single-cell transcriptomics rather than unique to GRAIL-Heart, but it affects interpretation.

### Future improvements

The following enhancements are planned to address identified limitations:

i. Multi-tissue pretraining: Training on diverse tissues (immune, epithelial, vascular) to improve generalisation and reduce cardiac specificity bias.
ii. Lightweight model variant: Pruned or distilled version optimised for deployment on standard hardware (the current ∼12.3M parameter model is already suitable for standard GPU deployment; a further-distilled CPU-deployable version targeting <5M parameters would aid in resource-constrained clinical settings)
iii. Temporal extensions: Modifications to handle time-series spatial transcriptomics, predicting interaction dynamics
iv. Uncertainty quantification: Bayesian versions or ensemble approaches providing confidence intervals rather than point estimates
v. Active learning integration: User feedback loop allowing experimental results to iteratively improve predictions
vi. Database expansion: Automated integration of newly published L-R interaction data

## Ethics statements

This study analysed publicly available data from the Human Heart Cell Atlas v2, which was previously collected and processed under appropriate ethical approvals documented in the original publication by Kanemaru et al., 2023[12]**.** No new human subjects were enrolled or sampled for this study. All data analysed are publicly available and do not contain personally identifiable information.

No animal experiments, human subject recruitment, or primary tissue collection was performed. Method development involved only computational analysis of previously published, de-identified dataset. No ethical concerns specific to this computational framework.

## Web explorer/resource availability

The network explorer is available at https://tumo505.github.io/GRAIL-Heart/outputs/cytoscape/index.html. A full-featured web application supporting dataset upload, interactive L-R network exploration, and causal score visualisation is included in the app/ directory of the repository and is documented at https://tumo505-grail-heart.mintlify.app/introduction, the web app can be accessed at https://huggingface.co/spaces/Tumo505/grail-heart. Docker deployment is supported via the included docker-compose.yml for reproducible local installation (https://github.com/Tumo505/GRAIL-Heart/blob/main/docker-compose.yml). A compiled python package for easy installation can also be accessed at https://pypi.org/project/grail-heart/1.0.0/.

## Data availability and reproducibility

All code and trained models are made available under open-source licenses (Apache-2.0) to maximise reproducibility and benefit to the research community. Source code, documentation, and analyses results are provided at https://github.com/Tumo505/GRAIL-Heart.

## Funding

This research did not receive any specific grant from funding agencies in the public, commercial, or not-for-profit sectors.

## Declaration of competing interest

The authors declare that they have no known competing financial interests or personal relationships that could have appeared to influence the work reported in this paper.
